# Notes on the Genus *Aceratoneuromyia* Girault (Hymenoptera: Eulophidae) †

**DOI:** 10.3390/insects13050450

**Published:** 2022-05-10

**Authors:** Ning Huangfu, Huan-Xi Cao, Chao-Dong Zhu

**Affiliations:** 1 Key Laboratory of Zoological Systematics and Evolution, Institute of Zoology, Chinese Academy of Sciences, Beijing 100101, China; huangfuning@ioz.ac.cn (N.H.); zhucd@ioz.ac.cn (C.-D.Z.); 2 College of Biological Sciences, University of Chinese Academy of Sciences, Beijing 100049, China; 3 National Animal Collection Resource Center, Institute of Zoology, Chinese Academy of Sciences, Beijing 100101, China

**Keywords:** Chalcidoidea, new taxon, parasitoid, dipteran host, DNA barcoding

## Abstract

**Simple Summary:**

Fruit flies in the family Tephritidae are economically important pests of edible fruits, with some known hymenopteran parasitoids. Although *Aceratoneuromyia* *indica* (Silvestri) is one of the most common parasitoids and has been used in biological control of fruit flies, its establishment in biocontrol is impeded by the difficulties of proper identity. The genus *Aceratoneuromyia* Girault (Hymenoptera: Eulophidae) is studied here, and the generic name *Trjapitzinichus* Kostjukov and Kosheleva is proposed as a new synonym under *Aceratoneuromyia* as well. Based on morphology and DNA barcodes, *A. bilinis* Huangfu and Cao sp. nov., *A. carinata* Cao and Zhu sp. nov. and *A. trilinus* Cao and Zhu sp. nov. are described and illustrated. The well-known parasitoid of fruit flies, *A. indica*, is treated here with the diagnosis and illustrations. In addition, this study provided a morphological diagnosis for *Aceratoneuromyia* as well as a key to world species of this genus. We also briefly discussed the relationship of *Aceratoneuromyia* with other possibly close groups based on available morphological data. Together with DNA barcodes of *A. bilinis* sp. nov. and *A. indica* generated here, this study provided essential and useful information for the species identity of *Aceratoneuromyia*.

**Abstract:**

Fruit flies in the family Tephritidae are well known as economically important pests of edible fruits and can often cause serious damage and losses to both agriculture and the economy. One of the common parasitoids of fruit flies, *Aceratoneuromyia* *indica* (Silvestri), has been used in biological programs. However, the biocontrol utilities of parasitoids are impeded by the difficulties of proper identification. Species of the genus *Aceratoneuromyia* Girault (Hymenoptera: Eulophidae), usually developed as parasitoids of fruit flies, are studied here. *Trjapitzinichus* Kostjukov and Kosheleva is proposed as a new synonym under *Aceratoneuromyia*. Three new species of *Aceratoneuromyia*, *A. bilinis* Huangfu and Cao sp. nov., *A. carinata* Cao and Zhu sp. nov., and *A. trilinus* Cao and Zhu sp. nov., are described and illustrated from China. *Aceratoneuromyia indica* is also treated here with diagnosis and illustrations. DNA barcodes of *A. bilinis* and *A. indica* and a key to the world species of *Aceratoneuromyia* are provided. This study provided important identification information of parasitoids with morphology and molecular evidence, which is useful for imperative needs regarding the identity of parasitoids attacking fruit flies.

## 1. Introduction

Many members of dipteran flies are of great economic importance, and some of them are serious pests in agriculture. Among these pests, some species of the family Tephritidae known as fruit flies attack mostly mature or maturing edible fruits and can make serious fruit infestation and cause significant economic losses, such as Oriental fruit fly, *Bactrocera dorsalis* (Hendel), and medfly, *Ceratitis capitata* (Wiedemann) [1,2]. There are a few projects on the fruit fly parasitoids [3,4,5], but the utilities of these parasitoids in the field release were possibly not well established as reported before [2,6]. *Aceratoneuromyia indica* (Silvestri), which is developed as a gregarious and koinobiont endoparasitoid, has been widely reported as a successful biological control agent against fruit flies worldwide [7,8]. It has been also recorded in China [1]. However, to some extent, the colony establishments of parasitoids used for field release are hindered by difficulties of identification and little knowledge of their biology.

Species of *Aceratoneuromyia* Girault have been known as primary gregarious endoparasitoids of Diptera, mainly Anthomyiidae and Tephritidae, or as hyperparasitoid (e.g., *A. granularis* Domenichini) of Lepidoptera (*Phragmites australis* (M.C. Day) through parasitic Sarcophagidae of Diptera (e.g., *Cleigastra apicalis* (Meigen)) [7,9]. This genus is one of several small genera in the subfamily Tetrastichinae (Hymenoptera: Eulophidae), with ten described species before the establishment of *Trjapitzinichus* Kostjukov and Kosheleva [7,9,10]. Graham [7] revised the European species of *Aceratoneuromyia* and provided keys to the females of seven species and males of five species. Kostjukov and Kosheleva [10] established a new genus *Trjapitzinichus* to transfer three of these 10 known species, *A. evanescens* (Ratzeburg), *A. lakica* Kostjukov and Gunasheva, and *A. polita* Graham, from *Aceratoneuromyia* to *Trjapitzinichus*, which are included as valid in the Universal Chalcidoidea Database (UCD) [9]. Another two species of *Trjapitzinichus* described based on only holotype by Kostjukov and Kosheleva [11] were not included in UCD. Therefore, seven species have been included in the genus *Aceratoneuromyia* and five species in *Trjapitzinichus* [9] before this study. However, the validity of *Trjapitzinichus* and its relationship with *Aceratoneuromyia* remains elusive based on morphology only, which requires further confirmation by molecular evidence from at least these two groups and other closely related taxa. Before that, this study considers *Trjapitzinichus* as a junior synonym under *Aceratoneuromyia*.

Species of *Aceratoneuromyia* are known mainly from the Old World [7]. Most species of *Aceratoneuromyia* were described or recorded from Palearctic (Europe), and three were described from Oriental (India, Japan), which is hypothesized to result from two possible aspects. On the one hand, *Aceratoneuromyia* species have a natural distribution in Europe; on the other hand, Europe has more taxonomists working on Chalcidoidea, increasing the possibilities of discovering species of *Aceratoneuromyia*. However, the European species *A. fimbriata* Graham has been recorded from the New World and *A. indica* also has been widely spread throughout the world as a biological control agent of fruit flies [8,9]. Despite their importance as a parasitic group and occupying an important niche in the ecosystem, it remains unclear about the phylogenetic relationships of *Aceratoneuromyia* with the other genera in Tetrastichinae. LaSalle [8] inferred that the similarities between *Aceratoneuromyia* and *Melittobia* are possibly due to convergence based on the available evidence at that time. 

This study aims to describe three new species of *Aceratoneuromyia* and synonymize the name *Trjapitzinichus* with *Aceratoneuromyia* and discuss the possible relationships of *Aceratoneuromyia* with other possibly close genera in Tetrastichinae based on morphology. Although DNA barcode fragments of two species of *Aceratoneuromyia* (*A. bilinis* and *A. indica*) were generated in this study, by referring to descriptions and specimens of *Aceratoneuromyia* species, this study summarized the concept of *Aceratoneuromyia*. Clear classification and identification of these important parasitoids will facilitate their practical applications in agriculture as potential biocontrol agents and help to better understand interactions with their hosts and other organisms from evolutionary and ecological perspectives. 

## 2. Materials and Methods

### 2.1. Insect Collection and Rearing

The live larvae of *Pelmatops ichneumoneus* were originally collected during field trips in Yunnan, China, 2020 and reared in the laboratory by feeding on its host plant *Rubus multibracteatus* Levl. et Vant. (Rosaceae), which was transplanted from the collection locality in Yunnan. Larvae and pupae of *Bactrocera dorsalis* were lab-reared and originally collected from field in Fujian, China. The host pupae were placed in plastic cups with a mesh cloth cover and moved to the laboratory of the Institute of Zoology, Chinese Academy of Sciences (IZCAS) to rear adults of fruit flies and parasitoid wasps. Emerged wasps were then preserved in 100% ethanol for further use after emergence.

### 2.2. Specimens Preparations

Specimens were examined with a Nikon SMZ 1500 stereomicroscope fitted with a 10 mm ocular grid having 100 divisions. Some ethanol-preserved specimens of parasitoid wasps were critical-point dried with a Leica EM CPD300 automated critical point dryer for morphological studies. Several critical-point dried specimens were dissected into head, mesosoma, and metasoma for scanning electron microscopy (SEM), which were subsequently sputter-coated with gold using a Leica EM SCD050 super cool sputter coater. Micrographs were produced using an FEI Quanta 450 environmental scanning electron microscope. Habitus pictures were taken with a Nikon D7000 digital camera connected to the Nikon SMZ 1500 stereomicroscope and then stacked in Helicon Focus software to generate single highly focused images. All images were processed with Adobe Photoshop CC 2019.

Morphological terminology follows Gibson [12]. Abbreviations used for morphological characters are as follows: F1–F3, funiculars 1–3; C1–C3, clavomeres 1–3; MLM, midlobe of mesoscutum; Gtn, gastral tergite number; POL, the shortest distance between the posterior ocelli; OOL, the shortest distance between an eye and posterior ocellus. 

### 2.3. Molecular Analyses

Whole genomic DNA of 15 *Aceratoneuromyia* individuals (Table 1) was extracted using the DNeasy Blood & Tissue Kit (Qiagen GmbH, Hilden, Germany) following the manufacturer’s instructions. Two primer pairs, LCO1490 (5′-GGTCA ACAAA TCATA AA-GAT ATTGG-3′) [13] and HCOout (5′-CCAGG TAAAA TTAAA ATATA AACTTC-3′) [14], C1-J-1514 (5’-TATCA ACCAA TCATA AAGAT ATTGG-3’) [13] and C1-N-2194 (5’-CCCGG TAAAA TTAAA ATATA AAC-3’) [15], were used to amplify COI (mitochondrial cytochrome c oxidase I) sequences. All polymerase chain reactions (PCR) were conducted using LongGene T20 Multi-Block Thermal Cycler (Hangzhou, China) using La Taq polymerase (Takara, Japan) under the following conditions: initial denaturation for 1 min at 94 °C; 4 cycles of 1 min at 94 °C, 1.5 min at 45 °C, and 1.5 min at 72 °C; 35 cycles of 2 min at 94 °C, 1.5 min at 58 °C, and 1 min at 72 °C s; followed by a final extension at 72 °C for 5 min. Sequencing was performed in a single direction. COI sequences were examined and aligned in BioEdit version 7.0.9.0 [16]. The COI alignment matrix after trimming was translated into the amino acids in MEGA7 [17] to check for stop codons. A phylogenetic tree by the Kimura 2-parameter (K2P) model with Neighbor-Joining (NJ) method was constructed in MEGA 7.0 with 1000 bootstrap replicates to calculate support values for nodes. Voucher specimens are deposited in IZCAS. The COI sequences generated in this study have been deposited in GenBank under accession numbers ON260937–ON260951.

## 3. Results

During recent programs on the biology of Tephritidae in China, fresh materials of two species of *Aceratoneuromyia* were respectively collected and reared from the pupae of *Bactrocera dorsalis* (Hendel) and *Pelmatops ichneumoneus* (Westwood) in the family Tephritidae. The species reared from *Bactrocera dorsali* was identified as *A. indica*, and the other species reared from *Pelmatops ichneumoneus* was identified as a species new to science. By comparing with specimens deposited in the insect collection of IZCAS, two additional new species of *Aceratoneuromyia* were discovered as well. As a result, three new species were treated and illustrated in this study.

**Figure 1:**
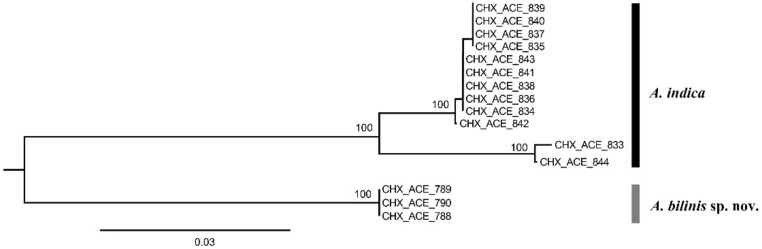
A NJ tree by K2P distances based on COI sequences from 15 *Aceratoneuromyia* specimens. The numbers above nodes represent bootstrap values.

Fifteen COI sequences were successfully sequenced from 3 females of *Aceratoneuromyia bilinis*, and 6 females plus 6 males of *A. indica*. A COI matrix with a length of 657 base pairs was obtained based on these 15 COI sequences after alignment and trimming, which has no insertion or deletion. A NJ tree (Figure 1) by K2P distances was generated based on this COI alignment. 

### 3.1. Morphological Diagnosis and Species Treatments of Aceratoneuromyia

The genus *Aceratoneuromyia* Girault, 1917

*Aceratoneuromyia* Girault, 1917 [18]: 151. Type species: *Aceratoneuromyia australia* Girault, by original designation.

*Trjapitzinichus* Kostjukov & Kosheleva, 2006 [10]: 108. Type species: *Entedon evanescens* Ratzeburg, by original designation. Syn. nov.

**Figure 2:**
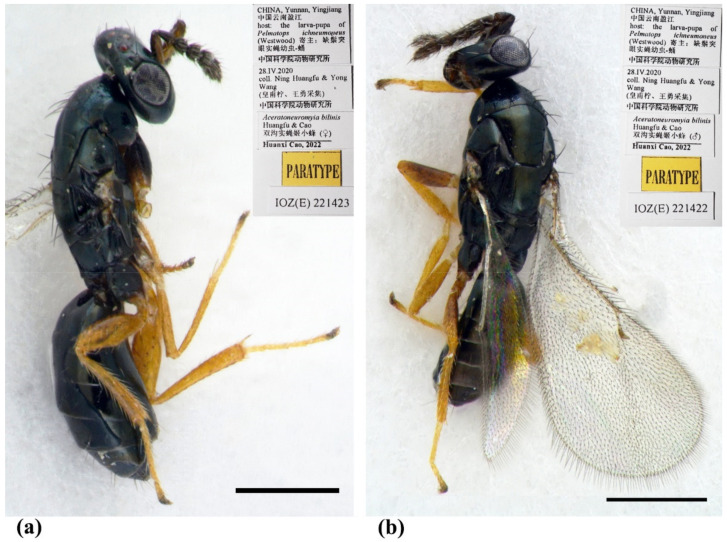
*Aceratoneuromyia bilinis* sp. nov. (**a**) Habitus of female (paratype, IOZ(E)221423) in dorsolateral view; (**b**) Habitus of male (paratype, IOZ(E)221422) in dorsolateral view. Scale bar: 0.5 mm.

**Figure 3:**
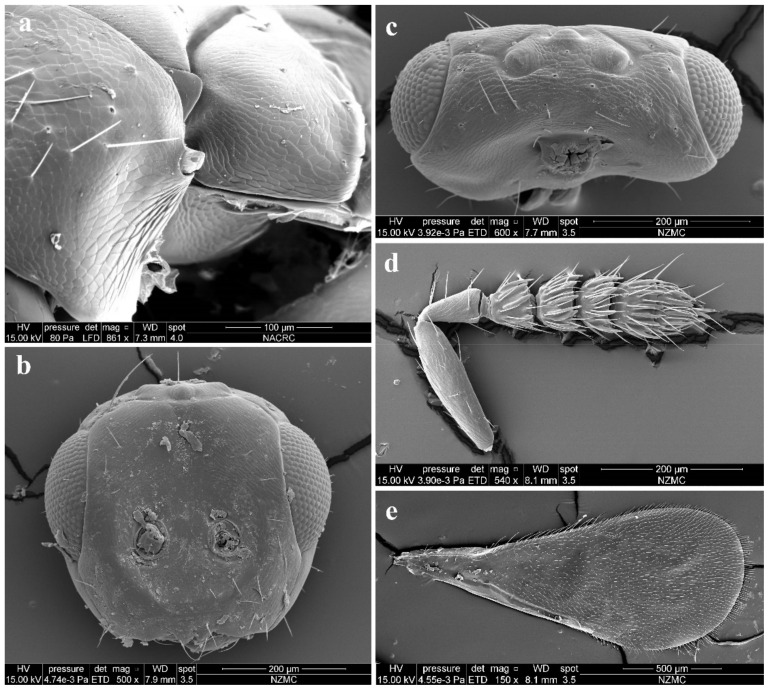
*Aceratoneuromyia bilinis* sp. nov., female. (**a**) Lateral spiracles of pronotum in dorsolateral view; (**b**) Face; (**c**) Head in dorsal view; (**d**) Antenna in lateral view; (**e**) Forewing.

**Figure 4:**
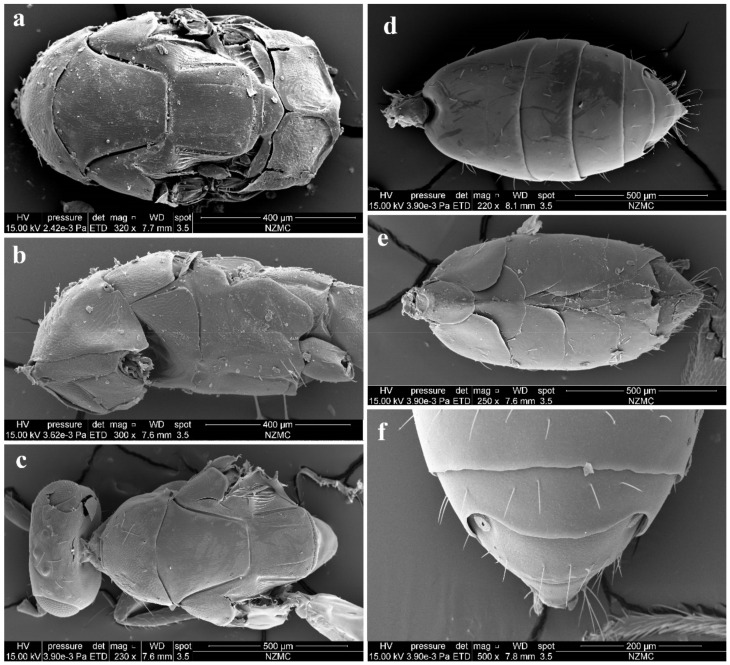
*Aceratoneuromyia bilinis* sp. nov., female. (**a**) Mesosoma in dorsal view; (**b**) Mesosoma in lateral view; (**c**) Head plus thorax in dorsal view; (**d**) Metasoma in dorsal view; (**e**) Metasoma in ventral view; (**f**) Gt_5–6_ in dorsal view.

**Figure 5:**
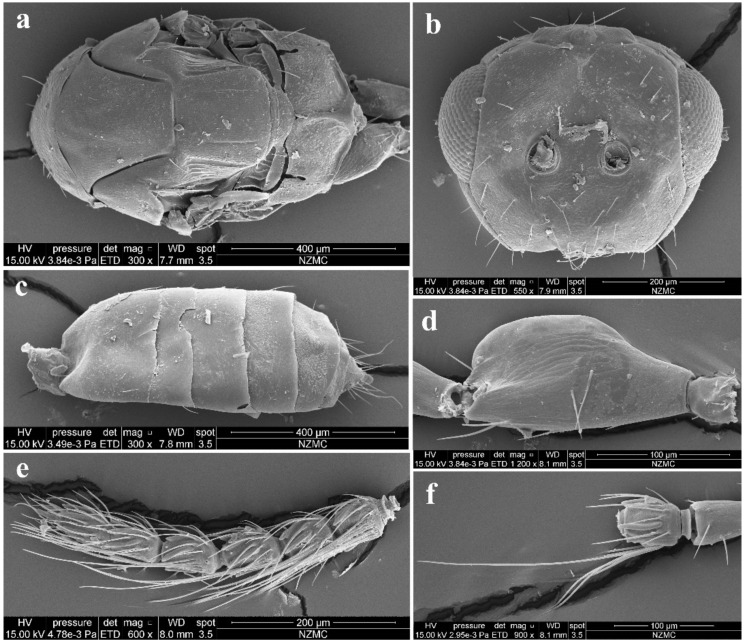
*Aceratoneuromyia bilinis* sp. nov., male. (**a**) Mesosoma in dorsal view; (**b**) Face; (**c**) Metasoma in dorsal view; (**d**) Scape of antenna; (**e**) Flagellum of antenna; (**f**) F1 with dorsal setae.

Diagnosis. Antenna with very long apical seta of terminal spine (about 2× as long as terminal spine, sometimes broken off) and with long, scattered erect or semi-erect setae (e.g., Figures 3d and 9b) as well as male antenna, with each funicular having a short transverse row of long setae on the dorsal surface (e.g., Figures 5e and 9h); female antenna with funiculars usually quadrate to transverse (e.g., Figure 9h), rarely distinctly longer than broad; male antennal scape usually with (e.g., Figure 3d) and rarely without (e.g., A. evanescens) ventral plaque. Malar sulcus present, from weak to groove-like. Frontofacial sutures (e.g., Figures 3b and 9a,g) not or only slightly divergent dorsally, sometimes absent. Scutellum with (e.g., Figure 9a) or without (e.g., Figure 9d,i) submedian lines, if present, then weak and incomplete, with sublateral lines distinct and laterally carinate (e.g., Figure 9a); scutellum with anterior pair of setae situated in the posterior half and close to the posterior pair of setae (e.g., Figure 9a,d,i). Gaster usually slender, rarely rounded, with ovipositor sheaths not visible even ventrally (e.g., Figures 4e and 9f,k); spiracles of penultimate segment of gaster rather large (relatively smaller in males), usually visible in dorsal view, rarely directed laterally and not visible in dorsal view (e.g., Figures 4f and 9e,j); female gaster usually tending to be convex dorsally (e.g., Figure 2a and Figure 8a). Hypopygium extending distinctly more than half length of the gaster, nearly to the apex (e.g., Figures 4e and 9f,k).

Remarks. The diagnosis of *Aceratoneuromyia* was listed here by referring to the three newly described species, descriptions of the other known species, as well as the diagnosis given by Bouček [19], Graham [7], LaSalle [8], and Ikeda [20]. Ikeda [20] has proposed a modified concept for *Aceratoneuromyia* with the addition of a new species from Japan and South Korea, *A. kamijoi* Ikeda, which disagrees in some characters with the concept previously given. This study follows those modifications to interpret the genus *Aceratoneuromyia*. Based on this genus concept of *Aceratoneuromyia* and re-descriptions of *Trjapitzinichus* by Kostjukov & Kosheleva [11], sometimes the placement of some intermediated species into one of them could be confusing. Furthermore, the separation of *Trjapitzinichus* from *Aceratoneuromyia* by Kostjukov & Kosheleva [10,11] obscures the differences between these two genera. It is hereby considered unreasonable to keep them as separate genera and *Trjapitzinichus* is synonymized with *Aceratoneuromyia* here.

#### 3.1.1. *Aceratoneuromyia bilinis* Huangfu & Cao, sp. nov.

Diagnosis. Female antenna clavate, with F1 subquadrate, F2 and F3 transverse (Figure 3d), with scape not reaching anterior ocellus (Figure 2a). Frontofacial sutures narrowly separated from each other and then passed as an extremely short line that just reaching shallow scrobes in frontal view (Figure 3b and Figure 5b), and divergent and visible dorsally, sometimes connecting the vertexal sutures (Figure 3d and Figure 5b). Pronotum with a row of 4 long erect setae near posterior margin (Figure 3a and Figure 5a). MLM with 3 adnotaular setae (Figure 3a and Figure 5a). Scutellum with somewhat distinct but incomplete submedian lines (Figure 3a and Figure 5a). Dorsellum large, about 2× as broad as high. Metasoma slender (Figure 2a,b, Figure 4d and Figure 5c), as long as the combined length of mesosoma and head; in female, convex dorsally in female (Figure 2a), dorsolateral posterior margin of Gt_5_ circled along outer margin of large spiracles of Gt_6_ (Figure 4f). The longest cercal seta 2× length of the other 3 setae equal in length and weakly curved apically (Figure 4e and Figure 5c).

Female (Figure 2a, Figure 3 and Figure 4). Body length 2.0–2.3 mm. Body black with a very weak dark coppery green tinge (Figure 2a), with tegula fuscous, with black setae. Brown antenna except scape and pedicel yellowish-brown ventrally (Figure 2a). Legs with fore and hind coxae dark brown, mid coxae brown, remainder parts testaceous except dark claws (Figure 2a). Wings hyaline, with brown veins.

Antenna (Figure 3d) with 3 funiculars and 3 clavomeres, setae of pedicel and flagellum standing out strongly; scape not reaching anterior ocellus, about 2.5× as long as pedicel; funicle proximally distinctly stouter than pedicel; pedicel distinctly longer than F1 (0.7:0.5); F1–F3 gradually decreasing in length (0.5:0.4:0.3) and increasing in width, F1 wider apically, length equal to apical width, F2 and especially F3 transverse; clava as long as F2 plus F3, clavomeres decreasing in length, C3 with a short but distinct terminal spine that has a distinctly long seta (about 2× length of terminal spine). Each flagellomere with longitudinal sensilla and apically with a circle of scattered, mushroom-shaped capitate peg sensilla; each flagellomere except C3 truncate apically.

Head (Figure 3b,c) slightly broader than mesoscutum in dorsal view (4.0:3.5), about 3× as broad as long (4.0:1.3); vertex with long and erect setae, and a vertex suture at each side. OOL as long as POL (0.70:0.70). Ocelli arranged in a strong obtuse triangle. Head in frontal view about 1.14× as broad as high (4.0:3.5). Face with extremely fine delicately engraved reticulation, much weaker than that of thorax. Frons with a short and narrow Y-shaped frontofacial suture, with V-shaped diverging lines narrowly separated from each other, extending to vertex dorsally and sometimes connecting vertex suture, and with extremely short vertical line reaching scrobes (Figure 3b,c). Toruli inserted slightly above lower margin of eyes. Eyes with few of short and white hairs, diameter larger than malar space (2.0:1.0). Malar space 0.4–0.5× length of mouth opening, and malar sulcus almost straight and weakly curved in lateral view. Anterior margin of clypeus bilobed (Figure 3b).

Pronotum long, sloping and visible in dorsal view, with distinct and engraved reticulation, neck and collar not delimited, without posterior carina; with some setae at sides and 4 long setae near posterior margin (Figure 4a). Mesoscutum with extremely fine delicately engraved and longitudinal reticulation having areoles 2× or less as long as broad, notaular grooves deep; MLM with 2–3 adnotaular setae, without median line, with posterior margin straight (Figure 4a). Axillae strongly advanced, with engraved reticulation in the anterior half and with strongly raised striations (like carinae) in the posterior half (Figure 4a,c). Scutellum flattened in profile, slightly broader than long (2.5:2.1), sculptured rather more finely than mesoscutum; submedian lines present, distinctly nearer to sublateral lines than to each other, enclosing a space about 2.0–2.2× as long as broad; however, submedian lines are often incomplete, superficially indicated basally and apically, with two pairs of setae subequal in length, anterior pair situated well behind the middle and close to posterior pair, and posterior pair situated near posterior margin; scutellum with a sloping frenum (Figure 4a). Dorsellum about 1.86× as broad as high (1.3:0.7), about 0.54× length of propodeum (0.7:1.3), faintly engraved reticulate along outer margins (sculptures invisible under stereomicroscope), almost smooth; slightly incised in the middle of posterior margin; lateral panel of metanotum with more or less longitudinal but irregular carinae in the anterior 3/5 and smooth in the posterior 2/5 (Figure 4a). Propodeum raised-reticulate, with reticulation much coarser than that of scutellum (Figure 4a); with a strong medina carina having a short sulcus anteriorly, median carina distinctly raised, broadening caudad and then extending laterally; with rim of spiracle invisible, covered by the lobe of callus, while with a hole at the same position of spiracle and a broad sulcus from anterior margin to the spiracle position, with weak paraspiracular carina that is difficult to discern under certain lights of stereomicroscope; callus with raised reticulation as coarse as that of median area of propodeum, with 1 erect seta only. Lateral panel of pronotum weakly reticulate, prepectus, mesepimeron and mesepisternum nearly smooth; acropleuron smooth; metapleuron with raised reticulation like propodeum (Figure 4b).

Petiole distinct, nearly as long as broad, but partially hidden under certain angles (Figure 2a and Figure 4d). Gaster (Figure 4d,e) slender, about 2× as long as broad and nearly as long as head plus mesosoma, based on the median length, the largest Gt_1_ 1.8× Gt_2_, Gt_2_ not clearly delimited from Gt_1_, Gt_2_ 2/3 length of Gt_3_, Gt_3_ as long as Gt_4_; sclerotized, convex dorsally, almost as hard as thorax and not easily collapsed; gastral tergites with superfacial engraved reticulation, almost polished under stereomicroscope even under SEM (Figure 4d); dorsolateral posterior margin of Gt_5_ circled along outer rim of distinctly large spiracle of Gt_6_, large spiracle visible and distinct in dorsal view (Figure 4f); Gt_7_ with 4 cercal setae, the longest seta weakly curved apically and about 2× as long as the other 3 setae that are subequal in length (Figure 4d–f). Tip of hypopygium situated at 0.8× length of gaster measured from the base; anterior margin of hypopygium truncate, posterior margin bidentate (Figure 4e).

Legs with coxa, femora, and tibia-engraved reticulate; hind coxae compressed with dorsal carina on the apical 2/3 (distinct under certain lights). Fore wing (Figure 3e) densely setose, speculum small, somewhat closed below, not extending below marginal vein; costal cell 10–12× as long as wide, with 19 short setae on underside; submarginal vein with 5 setae on dorsal surface; marginal vein distinctly longer than submarginal vein (4.8:2.5); postmarginal vein absent.

Male (Figure 2b and Figure 5). Body length about 2 mm (Figure 2b). Differs from female as follows. Antenna with 3 funiculars and 3 clavomeres (Figure 5e), with scape expanded and flattened, about 2.25× as long as broad, ventral plaque about 0.6× length of scape (Figure 5d); scape and pedicel brow, flagellum dark brown (Figure 2b); pedicel 1.6× as long as broad, about 0.5× length of scape and 1.4× length of F1; funicle nearly filiform, about as stout as pedicel; F1–F4 subequal in length, about 1.25× as broad as long; C1 and C2 subequal in length, C3 about 0.4× length of C2, spine 0.85× length of C3, with apical seta nearly 3× as long as the spine; clava slightly broader than funicle, about 2.6× as long as broad, shorter than F2 plus F3, F1–C2 with a short transverse row of dark and long setae on the dorsal surface (Figure 5e,f). Gaster less sclerotized, dorsolateral posterior margin of Gt_5_ not circled, Gt_6_ with spiracle much smaller, difficult to discern in dorsal view under lights of a stereomicroscope (Figure 5c). 

Etymology. The name *bilinis* refers to the submedian lines on scutellum.

Type material. Holotype, female, CHINA, Yunnan, Yingjiang, 2020, coll. Yong Wang & Ning Huangfu, host: the larva-pupa of *Pelmatops ichneumoneus* (Westwood) (IZCAS, IOZ(E)221421). Paratypes: 10 females and 3 males, same data as holotype (IZCAS, IOZ(E)221422–IOZ(E)221434).

Host. This species, a gregarious endoparasitoid, parasitizes the larva of Tephritidae host *Pelmatops ichneumoneus* (Westwood) and emerges from the pupa of *P. ichneumoneus*. 

Distribution. Oriental (China: Yunnan).

Remarks. Before synonymizing *Trjapitzinichus* with *Aceratoneuromyia*, like *A. kamijoi* Ikeda, *A. bilinis* is another intermediated species by having submedian lines although incomplete on scutellum, torulli slightly above the lower margins of eyes, and distinct malar sulcus. This confirms the necessary modifications for the concept of *Aceratoneuromyia* by Ikeda [20] and suggests that it is reasonable to synonymize *Trjapitzinichus* with *Aceratoneuromyia*. 

#### 3.1.2. *Aceratoneuromyia carinata* Cao & Zhu, sp. nov.

**Figure 6:**
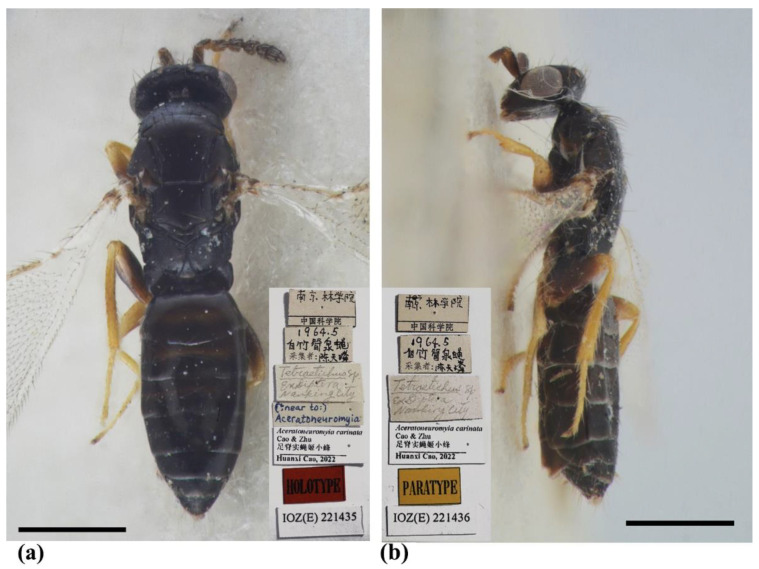
*Aceratoneuromyia carinata* sp. nov., female. (**a**) Habitus of the holotype in dorsal view; (**b**) Habitus of the paratype (IOZ(E)221436) in lateral view. Scale bar: 0.5 mm.

**Figure 7:**
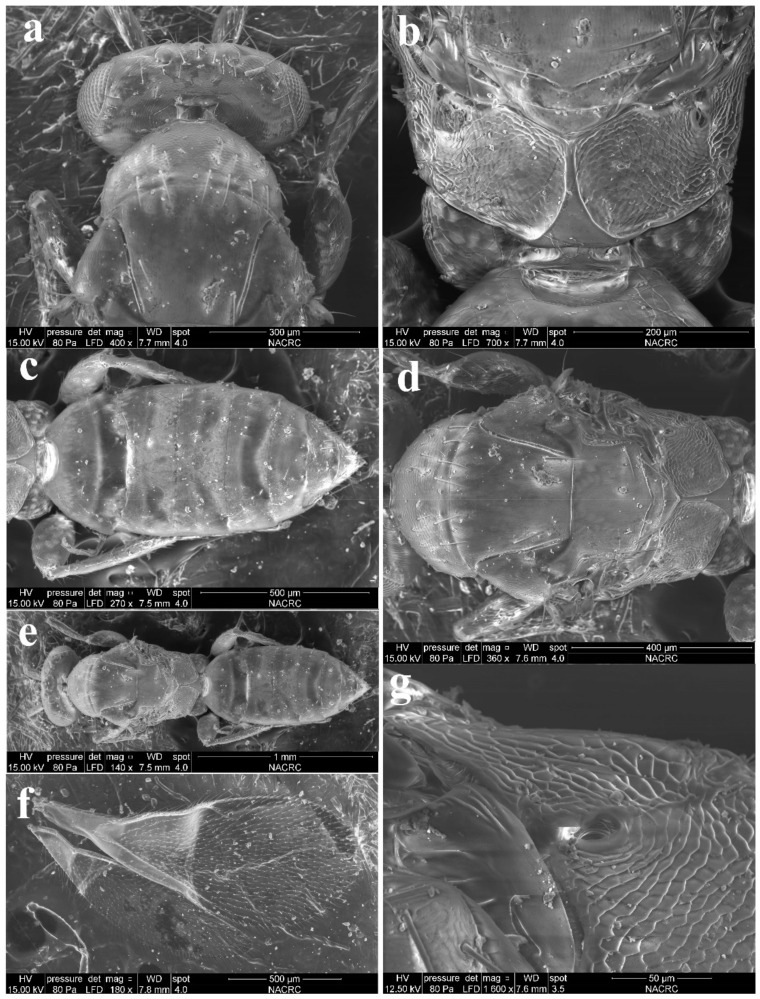
*Aceratoneuromyia carinata* sp. nov., female. (**a**) Head and pronotum in dorsal view; (**b**) Propodeum; (**c**) Metasoma in dorsal view; (**d**) Mesosoma in dorsal view; (**e**) Habitus in dorsal view; (**f**) Forewing; (**g**) Propodeal spiracle.

Diagnosis (female). Antenna with F1 longer than broad, F2–F3 subquadrate, with scape not reaching anterior ocellus. Frontofacial sutures broadly separated from each other and then passed as a line reaching deep scrobes in frontal view, and divergent and visible dorsally, connecting the vertexal sutures. Pronotum with a row of 7–8 long erect setae near posterior margin (Figure 7a). MLM with 3 adnotaular setae (Figure 7b). Scutellum with traces of incomplete submedian lines (Figure 6a and Figure 7d). Frenum of scutellum large, almost as long as dorsellum (Figure 7b). Gaster slender, slightly longer than the combined length of mesosoma and head, somewhat flattened dorsally, and dorsolateral posterior margin of Gt_5_ circled along outer margin of large spiracles of Gt_6_ (Figure 6a,b and Figure 7c). The longest cercal seta slightly longer than the other 2 setae equal in length, and very weakly curved apically (Figure 7c).

Female (Figure 6 and Figure 7). Body length 1.5–1.7 mm. Body dark brown without metallic tinge (Figure 6a,b), with tegula yellow, with pale setae. Antenna brown, with scape and pedicel yellowish ventrally, and flagellum paler ventrally. Legs with coxa, femora and claw brown, with remainder parts as well as trochanter, base and tip of femora yellow (Figure 6a,b). Wings hyaline, with brown veins.

Antenna with 3 funiculars and 3 clavomeres, pale setae of pedicel and flagellum standing out strongly; scape not reaching anterior ocellus, nearly 3× as long as broad (1.7:0.6), about 3.4× as long as pedicel (1.7:0.5); funicle stouter than pedicel, pedicel as long as F1 (0.5:0.5); each funicular equal in length (0.5:0.5:0.5), F1 longer than broad (0.5:0.4), F2–F3 subquadrate (0.5:0.5); clava slightly shorter than F2 plus F3 (1.2:1.0), C2 as long as but broader than C1, C3 with a short but distinct terminal spine. Each flagellomere with longitudinal sensilla and apically with a circle of scattered, mushroom-shaped capitate peg sensilla; each flagellomere truncate apically, except C3.

Head as long as mesoscutum in dorsal view (4.0:4.0), about 3× as broad as long (4.0:1.3); vertex with long and erect setae, with vertexal suture at each side. OOL as long as POL (0.6:0.6) (Figure 7a). Ocelli arranged in a strong obtuse triangle (Figure 7a). Head in frontal view about 1.33× as broad as high (4.0:3.0). Face with extremely fine delicately engraved reticulation, much weaker than that of thorax. Frons with a Y-shaped frontofacial suture, with diverging lines broadly separated from each other, extending to vertex dorsally and connecting vertex suture. Toruli inserted slightly below the lower margin of eyes, scrobes distinct and deep. Eyes with sparsely short and white hairs, diameter 2× as long as malar space (1.6:0.8). Malar space 0.5–0.6× length of mouth opening (0.8:1.5), and malar sulcus distinct and almost straight. Clypeus delimited laterally by pale line, with anterior margin bilobed.

Pronotum long, sloping and visible in dorsal view, distinctly engraved reticulate, neck and collar not delimited, without posterior carina; with some scattered setae and 6–8 long setae near posterior margin (Figure 7a). Mesoscutum with extremely fine delicately engraved and longitudinal reticulation, notaular grooves deep; MLM with 3 adnotaular setae, without median line, with posterior margin straight (Figure 7d). Axillae strongly advanced, with engraved reticulation anteriorly and with strongly raised striations (like carinae) in most posterior part (Figure 7d). Scutellum flattened in profile, distinctly broader than long (2.0:1.5), sculptured rather more finely than mesoscutum; with traces of incomplete submedian lines, superficially indicated basally and apically, distinctly nearer to sublateral lines than to each other, enclosing a space about 1.5× as long as broad (1.5:1.0); with two pairs of setae subequal in length, anterior pair situated well behind the middle and close to posterior pair, and posterior pair situated near posterior margin; scutellum apically with a moderately long frenum, as high as dorsellum (0.4:0.4), and not well differentiated from dorsellum (Figure 7a,d). Dorsellum about 2.75× as broad as high (1.1:0.4), about 0.54× median length of propodeum (0.4:1.3), faintly engraved-reticulate; slightly incised in the middle of posterior margin; lateral panel of metanotum with more or less longitudinal carinae in the anterior half and smooth in the posterior half (Figure 7b,d). Propodeum with distinct scale-like reticulation (Figure 7b,g); with a strong medina carina slightly raised, broadening caudad and then extending laterally; with rim of spiracle visible, propodeal spiracle separated by its diameter from metanotum; callus with raised reticulation more distinct than that of median area of propodeum, with 2 long erect setae and 2 short setae at post-lateral corner. Lateral panel of pronotum weakly reticulate, prepectus, mesepimeron and mesepisternum nearly smooth; acropleuron smooth; metapleuron with raised reticulation like propodeum.

Petiole transverse, hidden under a stereomicroscope (Figure 7b,c). Gaster (Figure 7c) slender, more than 2× as long as broad (8.2:3.8) and slightly longer than head plus mesosoma (8.2:8.0), based on the median length, the largest Gt_1_ about 1.83× Gt_2_ (2.2:1.2), Gt_2_ as long as Gt_3_ and Gt_4_ (1.2:1.2:1.2); alutaceous, with engraved reticulation; dorsolateral posterior margin of Gt_5_ circled along outer rim of distinctly large spiracle of Gt_6_, large spiracle visible and distinct in dorsal view; Gt_7_ with 3 cercal setae, the longest seta weakly curved apically and slightly longer than the other 2 setae that are subequal in length. Tip of hypopygium nearly reaching the apex of gaster, situated at 7/8 length of gaster measured from the base; anterior margin of hypopygium truncate, posterior margin bidentate.

Legs with coxa, femora, and tibia-engraved reticulate; hind coxae compressed with distinct and complete dorsal carina. Fore wing (Figure 7f) densely setose, speculum small, closed below, not extending below marginal vein; costal cell 10–12× as long as wide, with 7–8 short setae on underside; submarginal vein with 4–5 setae on dorsal surface; marginal vein distinctly longer than submarginal vein (4.0:3.2); postmarginal vein absent.

Male. Unknown.

Etymology. The name *carinata* refers to the complete dorsal carina on the hind coxa.

Type material. Holotype, female, CHINA, Jiangsu, Nanjing, Nanjing Forestry University, V.1964, coll. Tian-Lin Chen, host: *Pegomyia phyllostachys* Fan (IZCAS, IOZ(E)221435). Paratypes: 3 females, same data as holotype (IZCAS, IOZ(E)221436–IOZ(E)221438).

Host. This species was recorded from *Pegomyia phyllostachys* Fan (Diptera: Anthomyiidae), possibly from the host pupa as an endoparasitoid.

Distribution. Oriental (China: Jiangsu).

Remarks. *Aceratoneuromyia carinata*, along with the other two species, *A. claridgei* Graham and *A. granularis* Domenichini, has pronotum with a row of more than 4 long setae near posterior margin, while other species usually have a row of 4 long setae. However, *A*. *carinata* is readily differentiated from the other two species by the presence of submedian lines on the scutellum which seems unclear under SEM because old specimens were not sputter-coated with gold. Additionally, *A. carinata* has hind coxa with a complete dorsal carina and ventrally paler antenna.

#### 3.1.3. *Aceratoneuromyia indica* (Silvestri, 1910)

*Syntomosphyrum indicum* Silvestri, 1910 [21]: 230–244. Syntypes, USNM (not examined).

*Melittobia indicum* (Silvestri, 1910), Kurdjumov, 1913 [22]: 243–256.

*Aceratoneuromyia australia* Girault, 1917 [18]: 151. Syntypes, USNM (not examined). Synonymized by Gahan, 1938 [23]: 221.

*Aceratoneuromyia indica* (Silvestri, 1910), Gahan, 1938 [23]: 227.

**Figure 8:**
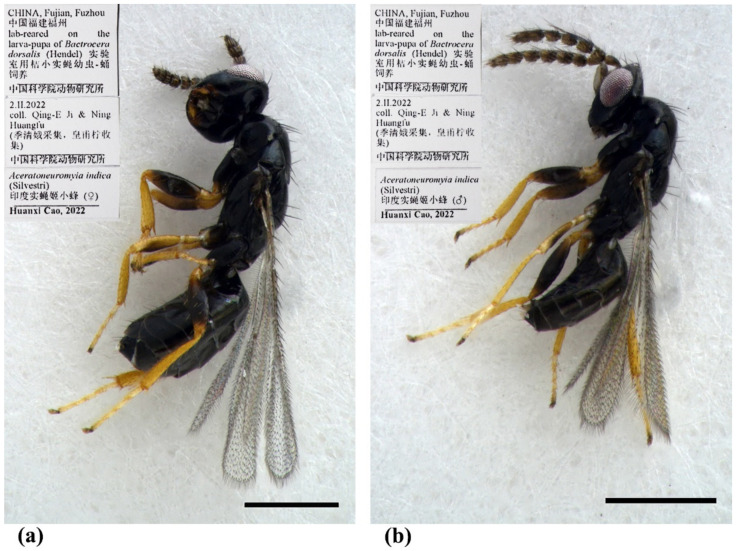
*Aceratoneuromyia indica* (Silvestri). (**a**) Habitus of female in lateral view; (**b**) Habitus of male in lateral view. Scale bar: 0.5 mm.

**Figure 9:**
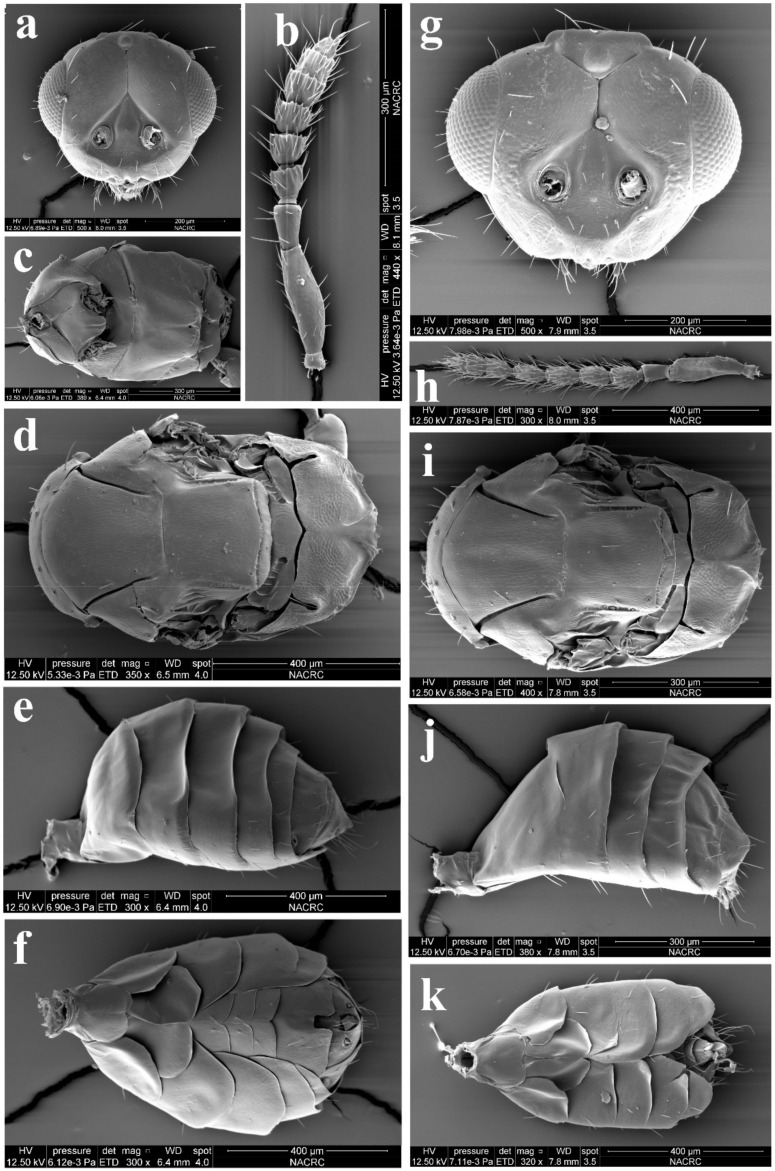
*Aceratoneuromyia indica* (Silvestri). Female: (**a**) Face; (**b**) Antenna; (**c**) Mesosoma in ventral view; (**d**) Mesosoma in dorsal view; (**e**) Metasoma in dorsolateral view; (**f**) Metasoma in ventral view; Male: (**g**) Face; (**h**) Antenna; (**i**) Mesosoma in dorsal view; (**j**) Metasoma in dorsolateral view; (**k**) Metasoma in ventral view.

Diagnosis. Female (Figure 8a) antenna clavate, with F1 somewhat as long as broad, with F2 and F3 somewhat more broad than long (Figure 9b), with scape not reaching anterior ocellus. Male scape with white ventral plaque about 0.8× length of scape (Figure 8b and Figure 9h). Frontofacial sutures broadly separated from each other and then passed as a line that reaching shallow scrobes in frontal view (Figure 9a,g), invisible dorsally because easily collapsed head. Pronotum with a row of 4 long erect setae near posterior margin (Figure 9d,i). MLM with 2 adnotaular setae subequal in length (Figure 9d,i). Scutellum without submedian lines (Figure 9d,i). Dorsellum distinct, about 2× as broad as high (Figure 9d,i). Gaster shorter than (Figure 9e) or as long as (Figure 9j) mesosoma, convex dorsally (Figure 9e,j). The longest cercal seta about 1.8× length of the other 3 setae equal in length and distinctly curved apically (Figure 9f,k).

Material examined. 10 females 8 males, CHINA, Fujian, Fuzhou, 2.II.2022, lab-reared from the pupae of *Bactrocera dorsalis* (Hendel), coll. Qing-E Ji & Ning Huangfu (IZCAS).

Host. This species can widely parasitize the larva of fruit flies in the family Tephritidae of Diptera [9].

Distribution. *Aceratoneuromyia indica* was originally described in India and now has been thought to be widely introduced as a biological control agent to all zoological regions [7,8,9]. However, currently, it is difficult to define its original distribution pattern based on occurrence records only.

#### 3.1.4. *Aceratoneuromyia* *trilinus* Cao & Zhu, sp. nov.

**Figure 10:**
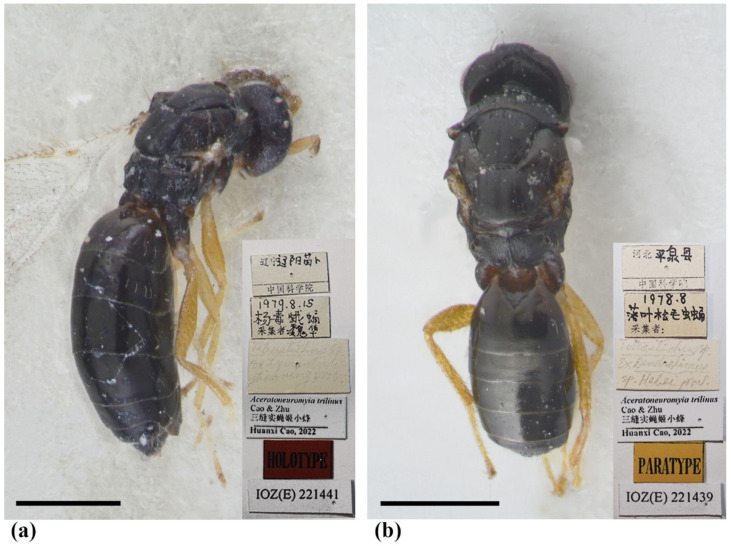
*Trjapitzinichus trilinus* sp. nov., female. (**a**) Habitus of the holotype in lateral view; (**b**) Habitus of the paratype (IOZ(E)221439) in dorsal view. Scale bar: 0.5 mm.

**Figure 11:**
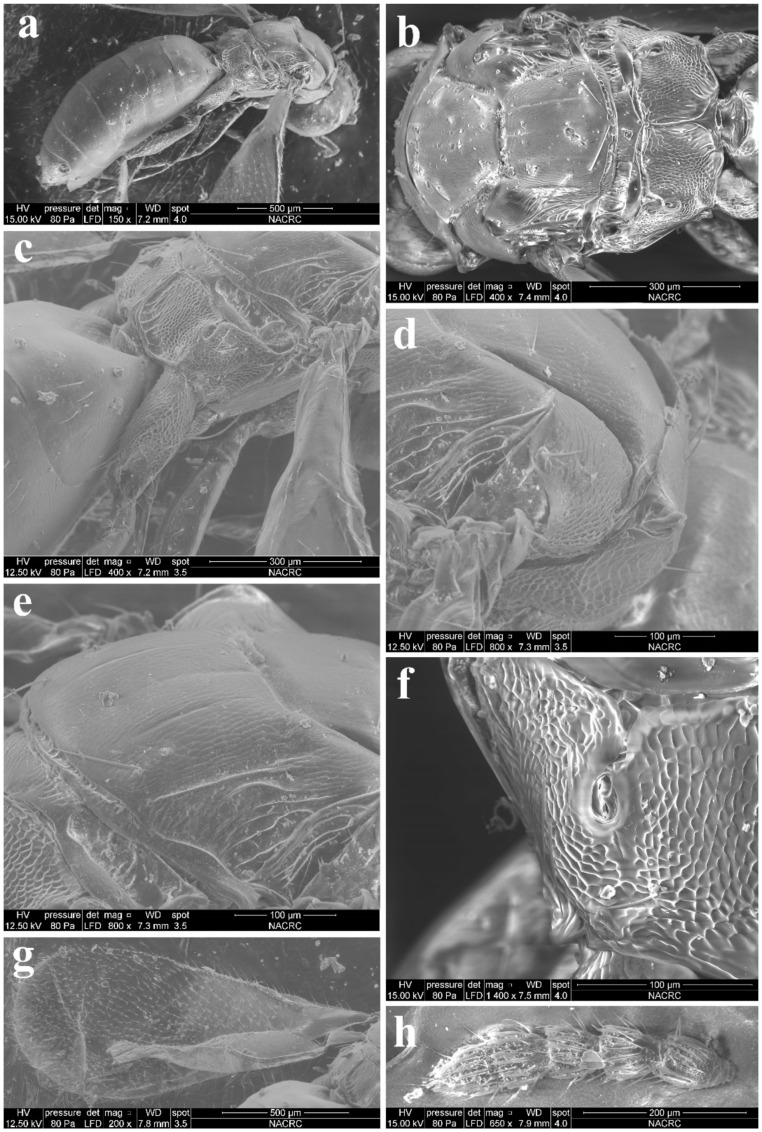
*Trjapitzinichus trilinus* sp. nov., female. (**a**) Habitus in lateral view; (**b**) Mesosoma in dorsal view; (**c**) Propodeum in lateral view; (**d**) Conical tubercle with spiracle in dorsolateral view; (**e**) scutellum in dorsolateral view; (**f**) Propodeal spiracle in dorsal view; (**g**) Forewing; (**h**) Flagellum in lateral view.

Diagnosis. Antenna with F1–F2 longer than broad, F3 subquadrate, with scape somewhat reaching anterior ocellus. Frontofacial sutures extremely short, almost absent, invisible in dorsal view; scrobes very shallow. Pronotum with a row of 4 long erect setae near posterior margin (Figure 11b). MLM with 2 adnotaular setae, with a trace of median line posteriorly (Figure 11b). Scutellum with incomplete submedian lines as well as a trace of median line in the anterior half (Figure 11b,e). Dorsellum very short, about 3× as broad as high (Figure 11b,c). Gaster slender, longer than the combined length of mesosoma and head (Figure 10a), dorsolateral posterior margin of Gt_5_ circled along outer margin of large spiracles of Gt_6_ (Figure 11a). The longest cercal seta 1.5× length of the other 3 setae equal in length and weakly curved apically.

Female. Body length 1.8–2.2 mm. Body dark brown without metallic tinge (Figure 10a,b), with pale setae. Antenna brown, with scape and pedicel paler dorsally and brownish yellow ventrally. Legs yellow, except for brown coxa and claw (Figure 10a). Wings hyaline, with brown veins.

Antenna with 3 funiculars and 3 clavomeres (Figure 11h), pale setae of pedicel and flagellum standing out strongly; scape somewhat reaching anterior ocellus, about 3× as long as pedicel (1.5:0.5), 3.78× as long as broad (1.5:0.4); funicle as stout as pedicel; pedicel, F1 and F2 equal in length (0.6:0.6:0.6) and longer than broad (0.6:0.5), F3 subquadrate (0.5:0.5); clava slightly shorter than F2 plus F3 (1.0:1.1), C1 longer than broad (0.7:0.5) and longer than F3 (0.7:0.5), C3 short, nearly as long as terminal spine. Each flagellomere with longitudinal sensilla and apically with a circle of scattered, mushroom-shaped capitate peg sensilla; each flagellomere except C3 truncate apically.

Head nearly as broad as mesoscutum in dorsal view (4.0:4.2), about 3.08× as broad as median length (4.0:1.3); vertex with long and erect setae, with weak vertexal suture at each side. OOL as long as POL (0.70:0.70). Ocelli arranged in a strong obtuse triangle. Head in frontal view, about 1.25× as broad as high (4.0:3.2). Face with extremely fine delicately engraved reticulation, much weaker than that of thorax. Frons with extremely short frontofacial suture, almost absent. Toruli inserted slightly above the lower margin of eyes. Eyes with sparsely short and white hairs, almost bare, diameter 1.8× as long as malar space (1.8:1.0). Malar space 0.5× length of mouth opening (1.0:2.0), with malar sulcus distinct and almost straight. Anterior margin of clypeus bilobed (Figure 3h). Pronotum long, sloping and visible in dorsal view, with distinct and engraved reticulation, neck and collar not delimited, without posterior carina; with some scattered setae and 4 long setae near posterior margin (Figure 11b); with spiracle on strong and distinct conical tubercle laterally (Figure 11d). Mesoscutum with extremely fine delicately engraved and longitudinal reticulation, notaular grooves deep; MLM with 2 adnotaular setae, with posterior margin straight (Figure 11b). Axillae strongly advanced, with engraved reticulation in about the anterior half and with strongly raised striations (like carinae) in about the posterior half (Figure 11c,d). Scutellum more or less flattened in profile, distinctly broader than long (2.5:2.0), sculptured rather more finely than mesoscutum; with traces of incomplete submedian lines, superficially indicated basally and apically, distinctly nearer to sublateral lines than to each other, enclosing a space about 2.3–2.5× as long as broad (2.5:1.0), as well as with a trace of median line in the anterior half; with two pairs of setae subequal in length, anterior pair situated well behind the middle and close to posterior pair, and posterior pair situated near posterior margin; scutellum with an extremely short frenum (Figure 11b,e). Dorsellum about 3× as broad as high (1.5:0.5), about 0.5× length of propodeum (0.5:1.0), faintly engraved-reticulate; slightly incised in the middle of posterior margin; lateral panel of metanotum with more or less longitudinal but irregular carinae in the anterior 4/5 and smooth in the posterior 1/5 (Figure 11c). Propodeum raised-reticulate, with reticulation slightly coarser than that of scutellum (Figure 11c,f); with a strong medina carina distinctly raised, broadening caudad and then extending laterally; with rim of spiracle visible, propodeal spiracle separated by 0.5–0.8 its diameter from metanotum; callus with raised reticulation as coarse as that of median area of propodeum, with 3 long erect setae and 1 long seta at post-lateral corner, not at the same plane with median area because of elevated median area (Figure 11f). Lateral panel of pronotum weakly reticultate, prepectus, mesepimeron and mesepisternum nearly smooth; acropleuron smooth; metapleuron with raised reticulation like propodeum.

Petiole transverse (1.0:0.2) (Figure 11c). Gaster slender, about 2.64× as long as broad (9.5:3.6) and slightly longer than head plus mesosoma (9.5:7.5) (Figure 10a), based on the median length, the largest Gt_1_ 1.25× Gt_2_ (2.5:2.0), Gt_2_, Gt_3_ and G_4_ equal in length; Gt_1_ smooth, the other tergites with superfacial engraved reticulation; dorsolateral posterior margin of Gt_5_ circled along outer rim of distinctly large spiracle of Gt_6_, large spiracle visible and distinct in dorsal view (Figure 11a); Gt_7_ with 4 cercal setae, the longest seta weakly curved apically and about 1.5× as long as the other 3 setae that are subequal in length. Tip of hypopygium almost reaching the apex; anterior margin of hypopygium truncate, posterior margin bidentate.

Legs with coxa (Figure 11c), femora, and tibia engraved-reticulate; hind coxae compressed with complete dorsal carina. Fore wing (Figure 11g) densely setose, speculum small, closed below, not extending below marginal vein; costal cell 10–12 × as long as wide, with 11 short setae on underside; submarginal vein with 4 setae on dorsal surface; marginal vein distinctly longer than submarginal vein; postmarginal vein absent.

Male. Unknown.

Etymology. The name *trilinus* refers to a median line and two submedian lines on the scutellum.

Type material. Holotype, female, CHINA, Liaoning, Liaoyang, Miaobu, 15.VIII.1979, host: the pupa of *Stilprotia salicis* (Linnaeus), coll. Hua Wei (IZCAS, IOZ(E)221441); Paratypes: 2 females, CHINA, Hebei, Pingquan, VIII.1978, host: the pupa of *Dendrolimus superans* (Butler) (IZCAS, IOZ(E)221439–IOZ(E)221440).

Hosts. Based on the label information of the above type material, two females were reared from the pupa of *Dendrolimus superans* (Butler) (Lepidoptera: Lasiocampidae), and one female was reared from the pupa of *Stilpnotia candida* Staudinger (Lepidoptera: Lymantriidae). The possibility that *A.* *trilinus* may be a hyperparasitoid of lepidopteran moths through other parasitoids is suggested by the host records of *A*. *evanescens* summarized in Noyes [9].

Distribution. Palaearctic (China: Hebei, Liaoning).

Remarks. *Aceratoneuromyia trilinus* is uniquely differentiated from the other known species in this genus by the trace of a median line on the scutellum.

### 3.2. Key to Species of Aceratoneuromyia

1. Pronotum subconical with distinct spiracles on conical tubercles; thorax weakly arched, propodeal slope as 15°–25° (e.g., Figure 10a and Figure 11a)…2 (previously under *Trjapitzinichus*)

- Pronotum without conical tubercles and with indistinct spiracles; flattened thorax, mesoscutum, scutellum and propodeum almost at the same plane (e.g., Figure 2 and Figure 8)…7

2(1) Pronotum with 2 long setae near its posterior margin; scutellum 2.15× as broad as long and with one pair of setae; propodeal spiracle separated by about 1.5× its diameter from metanotum; length of longest seta on vertex 1.3× maximum diameter of posterior ocellus; eye bare. Russia…*A. lakicus* (Kostjukov & Gunasheva)

- Pronotum with 4 long setae near posterior margin; scutellum 1.3–1.4× as broad as long, with two pairs of setae; propodeal spiracle separated by less than its diameter from metanotum; eye with short, sparse setae…3

3(2) Antennal scape somewhat longer than an eye, reaching anterior ocellus; pronotum bare, except for a row of 4 long setae near its posterior margin and sometimes 2–3 short ones at sides; propodeal callus polished, virtually smooth; metapleuron shiny but with weak alutaceous sculpture. Europe…*A. politus* (Graham)

- Antennal scape at most as long as an eye, not reaching anterior ocellus; pronotum with scattered short setae except for a row of setae near its posterior margin; propodeal callus and metapleuron distinctly alutaceous, sometimes with slightly raised reticulation…4

4(3) Scutellum with a trace of median line anteriorly (Figure 11e)…*A. trilinus* Cao & Zhu

- Scutellum without median line…5

5(4) Antenna with each funicular longer than broad; 3–4 tarsal segments dark; costal cell 15× as long as broad. North Korea…*A. leleji* Kostjukov & Kosheleva

- Antenna at least with F3 distinctly or slightly transverse; tarsal segments testaceous; costal cell at most 12× as long as broad…6

6(5) Eye 1.15× as long as broad; spine of clava 0.7× length of C3; pronotum 0.2× length of mesoscutum; MLM 1.45× as broad as long; propodeal spiracle separated by 0.25 its length from metanotum; tegula black; small species with body length 1.2 mm. Russia…*A. sugonjaevi* Kostjukov & Kosheleva

- Eye 1.3× as long as broad; spine of clava 0.5× length of C3; pronotum 0.3× length of mesoscutum; MLM about 1.3× as broad as long; propodeal spiracles separated by about 0.3 its length from metanotum; tegula fuscous; body length 1.4–1.6 mm. Germany…*A. evanescens* (Ratzeburg)

7(1) Females…8

- Males…16

8(7) Scutellum with submedian lines or with traces of submedian lines (e.g., Figure 3a)…9

- Scutellum without submedian lines (e.g., Figure 9d)…12

9(8) Scutellum only with traces of submedian lines (e.g., *Figure 9* in Ikeda [20]); torulli situated below the lower margin of eyes; frontofacial sutures distinct, scrobal grooves deep (e.g., *Figure 1* in Ikeda [20])…10

- Scutellum with somewhat distinct submedian lines although incomplete; torulus situated above the lower margin of eyes; frontofacial sutures absent or indistinct, scrobal grooves shallow (e.g., Figure 3b)…11

10(9) Vertex without vertexal sutures; pronotum with 4 long setae near posterior margin; propodeum with anterior part of median carina having narrow groove or small fovea; outer rim of spiracle partially covered by the raised lobe of callus; hind coxa without dorsal carina. Europe…*A. atherigonae* Ferriere

- Vertex with vertexal sutures; pronotum with 7–8 long setae near posterior margin; propodeum with anterior part of median carina without narrow groove or fovea; outer rim of spiracle not covered by the raised lobe of callus; hind coxa with complete dorsal carina (Figure 7b). China…*A. carinata* Cao & Zhu

11(9) Antenna with each funicular longer than broad; frontofacial sutures absent (*Figure 17* in Ikeda [20]); propodeal callus with 3–5 setae; spiracles of penultimate gastral tergite situated on lateral sides of gaster, invisible in dorsal view, Gt_5_ with posterior margin straight (*Figure 23* in Ikeda [20]). Japan, South Korea…*A. kamijoi* Ikedai

- Antenna with F2 and F3 transverse (Figure 3d); frontofacial sutures short and narrowed separated from each other and divergent on the vertex (Figure 3b,c); propodeal callus with 1 seta; spiracles of penultimate gastral tergite situated on dorsolateral sides of gaster, distinct and visible in dorsal view, Gt_5_ with posterior margin emarginated at dorsolateral corners (Figure 4f). China…*A. bilinis* Huangfu & Cao

12(8) Pronotum with a row of 8–12 long setae near posterior margin…13

- Pronotum with a row of 4 long setae near posterior margin…14

13(12) Pronotum with a row of 10–12 long setae near posterior margin; MLM with 3 adnotaular setae on each side; Larger species, 1.4–1.7 mm. UK…*A. claridgei* Graham

- Pronotum with a row of 8–10 long setae near posterior margin. MLM with 2–3 adnotaular setae on each side. Smaller species, length 1.1–1.4 mm. Europe…*A. granularis* Domenichini

14(12) F1 and F3 longer than broad, F2 subquadrate. Forewing with costal cell 21× as long as broad. Propodeam distinctly longer than scutellum. India…*A. wayanadensis* Narendran and Santhosh

- Antenna with each funicular transverse. Forewing with costal cell less than 14× as long as broad. Propodeam distinctly shorter than scutellum…15

15(14) Anterior adnotaular seta shorter than the posterior; tibiae infuscate with base and tip testaceous; forewing sparsely pilose all over, with costal cell more than 12× as long as broad. Czechoslovakia, USA…*A. filbriata* Graham

- Adnotaular setae equal in length; tibiae completely testaceous (Figure 8a); forewing tending to be thickly pilose all over, with costal cell less than 10× as long as broad. Worldwide…*A. indica* (Silvestri)

16(7) Antennal scape with ventral plaque only about 0.4 its length, situated in the upper half…17

- Antennal scape with ventral plaque 0.55–0.6× its length, extending somewhat into the lower half…18

17(16) Anterior adnotaular seta shorter than the posterior; tibiae infuscate with base and tips testaceous. Forewing sparsely pilose all over. Small species, length about 1 mm…*A. filbriata* Graham

- Adnotaular setae equal in length; tibiae completely testaceous (Figure 8b); forewing tending to be thickly pilose all over. Species tending to be larger (Figure 8b)…*A. indica* (Silvestri)

18(16) Scutellum with submedian lines or with traces of submedian lines…19

- Scutellum without submedian lines…21

19(18) Scutellum only with traces of submedian lines; torulli situated below the lower margin of eyes; vertex without vertexal sutures; frontofacial sutures distinct, scrobal grooves deep…*A. atherigonae* Ferriere

- Scutellum with distinct submedian lines; torulus situated above the lower margin of eyes; vertex with vertexal sutures; frontofacial sutures absent or indistinct, scrobal grooves shallow…20

20(19) Frontofacial sutures absent; propodeal callus with 3–5 setae…*A. kamijoi* Ikedai

- Frontofacial sutures short and narrowed separated from each other and divergent on the vertex (Figure 5b); propodeal callus with 1 seta (Figure 5a)…*A. bilinis* Huangfu & Cao

21(18) Pronotum with a row of 10–12 long setae near posterior margin; MLM with 3 adnotaular setae on each side; Larger species, 1.4–1.7 mm. UK…*A. claridgei* Graham

- Pronotum with a row of 8–10 long setae near posterior margin. MLM with 2–3 adnotaular setae on each side. Smaller species, length 1.1–1.4 mm. Europe…*A. granularis* Domenichini

## 4. Discussion

Kostjukov and Kosheleva [10] established the genus *Trjapitzinichus* to include three known species originally placed in *Aceratoneuromyia* and only summarized some differences with *Aceratoneuromyia* without argumentation. Kostjukov and Kosheleva [11] provided re-descriptions for *Trjapitzinichus*, and they did not mention and discuss the variations in some of those differences between *Aceratoneuromyia* and *Trjapitzinichus*. However, some intermediate species (e.g., *A. kamijoi* and *A. bilinis*) show some characteristics shared by both two genera, such as distinct malar sulcus, submedian lines of scutellum, toruli above the lower margin of eyes, and lateral projections on pronotum. These variations obscure the borderline between these two genera and bring difficulties in separating them. Those three species assigned to *Trjapitzinichus* are possibly just aberrant members of *Aceratoneuromyia* despite their lateral conical tubercles on the pronotum. Therefore, the generic name *Trjapitzinichus* is treated as a junior synonym of *Aceratoneuromyia* here although generic delimitations of most Tetrastichinae genera require confirmation from molecular evidence.

The phylogenetic relationships between *Aceratoneuromyia* and the other Tetrastichinae genera remain unclear. Gahan [23] synonymized *Aceratoneuromyia* with *Melittobia*, which were subsequently considered to be two distinct genera by other Eulophidae taxonomists [7,8,24,25]. Nevertheless, the affinity between *Aceratoneuromyia* and *Melittobia* was not supported by courtship behavior [26] and morphology which was briefly discussed by Cao et al. [27]. Although species of both *Tachinobia* (one of three genera in the *Melittobia* complex) and *Aceratoneuromyia* are gregarious parasitoids of some Diptera hosts, between which have no intersection, the former mostly in Tachinidae and the latter mostly in Tephritidae [8,28]. In addition, the *Melittobia* complex shows notable sexual morphological dimorphism, such as in the antenna, wings and eyes (males of *Koucoreckia* unknown) [27], whereas *Aceratoneuromyia* exhibits sexual difference restricted to only the antenna (e.g., Figure 9b,h). Therefore, the similarity between *Aceratoneuromyia* and the *Melittobia* complex is likely to be due to convergence, which requires further confirmation by comprehensive molecular evidence. Currently, it is still extremely hard to discuss the systematic position of *Aceratoneuromyia* as little is known about the phylogeny of Tetrastichinae. In contrast, *Aceratoneuromyia* is hypothetically more close to *Aprostocetus* than to *Tetrastichus*, with these two genera regarded as the two main lineages of Tetrastichinae.

## Figures and Tables

**Table 1:** Information of specimens sequenced with GenBank accession number of COI.

Code	Species	Sex	Host	Primer Pair	Accession Number
CHX_ACE_833	*A. indica*	female	*Bactrocera dorsalis*	LCO1490 & HCOout	ON260937
CHX_ACE_834	*A. indica*	female	*Bactrocera dorsalis*	LCO1490 & HCOout	ON260938
CHX_ACE_835	*A. indica*	female	*Bactrocera dorsalis*	LCO1490 & HCOout	ON260939
CHX_ACE_836	*A. indica*	female	*Bactrocera dorsalis*	LCO1490 & HCOout	ON260940
CHX_ACE_837	*A. indica*	female	*Bactrocera dorsalis*	LCO1490 & HCOout	ON260941
CHX_ACE_838	*A. indica*	female	*Bactrocera dorsalis*	LCO1490 & HCOout	ON260942
CHX_ACE_839	*A. indica*	male	*Bactrocera dorsalis*	LCO1490 & HCOout	ON260943
CHX_ACE_840	*A. indica*	male	*Bactrocera dorsalis*	LCO1490 & HCOout	ON260944
CHX_ACE_841	*A. indica*	male	*Bactrocera dorsalis*	LCO1490 & HCOout	ON260945
CHX_ACE_842	*A. indica*	male	*Bactrocera dorsalis*	LCO1490 & HCOout	ON260946
CHX_ACE_843	*A. indica*	male	*Bactrocera dorsalis*	LCO1490 & HCOout	ON260947
CHX_ACE_844	*A. indica*	male	*Bactrocera dorsalis*	LCO1490 & HCOout	ON260948
CHX_ACE_788	*A. bilinis*	female	*Pelmatops ichneumoneus*	C1-J-1514 & C1-N-2194	ON260949
CHX_ACE_789	*A. bilinis*	female	*Pelmatops ichneumoneus*	C1-J-1514 & C1-N-2194	ON260950
CHX_ACE_790	*A. bilinis*	female	*Pelmatops ichneumoneus*	C1-J-1514 & C1-N-2194	ON260951

## Data Availability

DNA sequence data are available on GenBank under accession numbers (ON260937–ON260951). Other data of this research were deposited in the Institute of Zoology, Chinese Academy of Sciences, Beijing, China.
